# Polymorphic Basal Rates of Continuous Subcutaneous Insulin Infusion among Taiwanese Children with Type 1 Diabetes

**DOI:** 10.1155/2015/250656

**Published:** 2015-01-31

**Authors:** Chia-Hung Lin, Feng-Ju Hsieh, Yang-Hau Van, Fu-Sung Lo

**Affiliations:** ^1^Division of Endocrinology and Metabolism, Department of Internal Medicine, Chang Gung Memorial Hospital and Graduate Institute of Clinical Medical Sciences, Chang Gung University, Taoyuan 333, Taiwan; ^2^Department of Pediatrics, Chang Gung Memorial Hospital and Chang Gung University College of Medicine, Taoyuan 333, Taiwan

## Abstract

*Introduction*. The basal dose of insulin, proportion of total daily insulin, and circadian variation during continuous subcutaneous insulin infusion (CSII) therapy among children with type 1 diabetes mellitus (T1D) have not been fully elucidated. *Materials and Methods*. A total of 45 childhood patients with T1D receiving CSII therapy at Pediatrics Department of Chang Gung Memorial Hospital between 2004 and 2012 were analyzed. Patients were classified according to Tanner stage. *Results*. HbA1c was significantly reduced in all Tanner groups within three months of CSII therapy (from 67 mmol/mol (8.3%) to 54 mmol/mol (7.1%), *P* < 0.05). The actual basal proportion of total daily insulin use was 34–40%. The circadian distribution of basal insulin differed markedly between the five Tanner groups. Basal insulin requirement was highest between 3:00 and 7:00 h in Tanner stages 1-2. In stages 3-4, a lower nocturnal basal insulin that increased gradually until daytime was noted. Adolescents (stage 5) displayed a high insulin peak between 6:00 and 11:00 h, and a smaller peak between 19:00 and 23:00 h. *Conclusions*. A smaller proportion of basal insulin to total daily insulin use, as well as varied circadian patterns of insulin use, characterized these children with T1D.

## 1. Introduction

The use of continuous subcutaneous insulin infusion (CSII) is becoming increasingly common within the pediatric population. In 2006, over 35,000 patients under the age of 21 years received insulin therapy through a pump system [1]. A number of challenges unique to caring for children and adolescents with type 1 diabetes (T1D) differentiate pediatric from adult care, notably the size of pediatric patients, and the presence of developmental issues in this population. Accordingly, the management of a child with type 1 diabetes must take into account the age and developmental maturity of the patient. Indeed, insulin requirement is based upon the body weight, age, and pubertal stage of the child. Not surprisingly, it is very difficult to consistently maintain euglycemia in most patients.

Prepubertal children usually require lower insulin doses, while higher doses are needed in pubertal children. The basal insulin requirement in children from Western countries is approximately 40 to 50% of the total daily dose [2]. However, we previously showed that basal insulin use is less than 40% of the total daily dose among young adult T1D patients in Taiwan [3]. In addition, there is a wide range of insulin to carbohydrate ratios, with more insulin being required in old children but less in young children than indicated by the calculations.

A recent study has revealed that patient age is the primary factor influencing both total daily requirement and circadian distribution of basal insulin when using CSII [2]. Puberty consists of a series of predictable events, and the sequence of changes in secondary sexual characteristics has been categorized by several groups. The staging system of puberty utilized most frequently was published by Marshall and Tanner [4, 5]. Differences in CSII dose distribution across Tanner stages remain unclear.

To clarify these issues, we designed a study of CSII treatment outcome assessment in pump-naïve type 1 diabetes pediatric patients in Taiwan.

## 2. Materials and Methods

We analyzed data from a total of 45 type 1 diabetic patients receiving CSII therapy at the Department of Pediatrics of Chang Gung Memorial Hospital between 2004 and 2012. In order to be included in the current study, participants had to meet all the following criteria: (a) very low C-peptide level (<0.35 ng/mL) [3, 6] with or without the presence of diabetic ketoacidosis, (b) pump-naïve, and (c) ambulatory and willing to cooperate during the program. Patients were treated with multiple daily injections of insulin glargine (Sanofi Aventis) or detemir (Novo Nordisk) as basal and insulin aspart (Novo Nordisk) as bolus at least three times daily. Patients were classified according to Tanner stage [4, 5]. The study was approved by the institutional review board and informed consent was obtained from the parents or guardians as needed.

The MiniMed Paradigm 712 pump (Medtronic MiniMed Inc., Northridge, CA, USA) was used for CSII. Each patient was admitted to the hospital and received instruction regarding intensive insulin adjustment. Capillary blood glucose was measured eight times daily for purposes of insulin dosing titration, including three measurements during the pre- and postprandial periods each, once at bedtime and at 0300 hours. The medical team, which comprised of diabetologists, educators, and dieticians, was continuously on-call to manage any unexpected conditions. All patients were carefully instructed on correct operation of the insulin pump until they achieved proficiency. Following discharge, all participants were followed up at outpatient clinics every 12 weeks for measurement of plasma HbA1C levels, lipid profiles, and renal and liver function tests.

The insulin regimen was switched from MDI to CSII according to a previously described protocol [3]. Briefly, the MDI total daily insulin dose was reduced by 25% to achieve a starting dose of CSII that would limit the possibility of hypoglycemia. Half of the insulin dose was infused continuously as the basal dose while the other half was divided into bolus doses for each meal and snack. The basal insulin dose was then titrated precisely as small as 0.1 U per hour to maintain the blood glucose targets in the range of 5–6.7 mmol/L from bedtime through the nocturnal period and 3.9–7.8 mmol/L before each meal. The bolus insulin dose was titrated up or down cautiously with 1 U every time for the fixed carbohydrate amount to keep postprandial glucose <8.3 mmol/L. The total daily dose of insulin was defined as the sum of basal and bolus insulin doses. The change of TDD (U/kg) was defined as the difference between prepump and postpump TDD ((prepump TDD–postpump TDD)/prepump TDD × 100%). Bolus insulin dose was defined as the sum of daily premeal insulin aspart doses.

Differences between groups in continuous variables were tested using a paired Student's *t*-test and ANOVA, as appropriate. Differences in proportions were assessed using a Chi-square or Fisher's exact test, as appropriate. Results were expressed as means ± standard deviation or numbers (%). The level of statistical significance was set at a *P* value of ≤0.05. Forty-five participants per treatment would provide 0.90 power. Statistical analyses were conducted with SAS (v9.3, SAS Institute, Cary, NC, USA).

## 3. Results

The distribution of the patients across the different Tanner stages is shown in Table 1. While the female percentage varied between 33 and 67% among the Tanner stage groups, no significant difference between groups was found. As expected, body weight, height, and body mass index (BMI) increased with advancing Tanner stages (*P* < 0.001). The standard deviation scores (SDS) of body weight, height, and body mass index (BMI) were not different across the Tanner stages. The average duration of diabetes was less than 6 years. In response to insulin therapy, average HbA1c levels fell between 53 and 75 mmol/mol (7–9%) with no significant difference across the stages. There was no evidence of diabetic retinopathy, nephropathy, and neuropathy among the participants.

**Figure 1:**
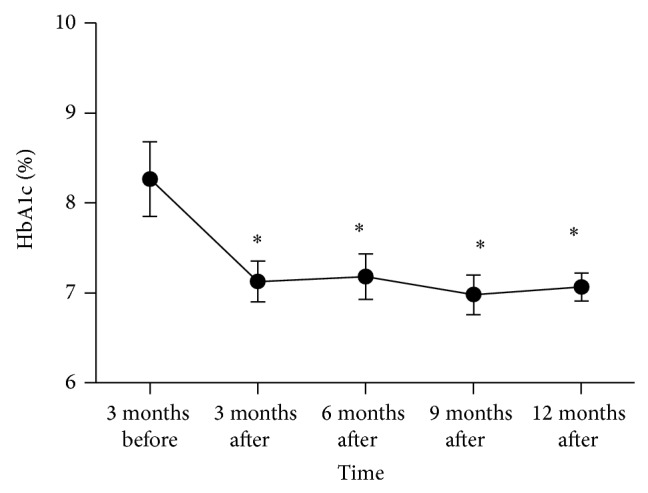
HbA1c change during continuous subcutaneous insulin infusion (CSII) therapy. ^*^ *P* < 0.05 as compared with the value 3 months before CSII therapy by paired *t* test. Data as mean ± standard deviation.

The patients' HbA1c levels were assayed before and every three months following initiation of the CSII program (Figure 1). The average baseline HbA1c level was 67 mmol/mol (8.3%) and decreased to 54 mmol/mol (7.1%) within three months of starting CSII treatment (*P* = 0.003, Table 2). The HbA1c levels were maintained at 53 mmol/mol (7%) during the one year of follow-up. The other clinical variables that changed during CSII therapy are listed at Table 2. The average fasting sugar values decreased significantly from 9.8 to 7.0 mmol/L (*P* = 0.026) as did the serum log transformed triglyceride levels (*P* = 0.013). Observed improvements in lifestyle included increased frequency of self-monitoring of blood glucose (SMBG; 4 to 6 times per day, *P* < 0.001) and duration of exercise (90 to 131 minutes per week, *P* < 0.001).

As shown in Table 3, the total daily insulin (U/kg) requirement did not differ significantly according to Tanner stage. Following CSII therapy, a decreased total daily insulin amount was documented among all Tanner stage groups (range: 10.8–25.4% reduction, *P* ≤ 0.05 for all stages, stage 5 and young adult groups). The basal portion of insulin ranged between 33.6 ± 4.7% and 39.6 ± 6.4% of the total daily insulin requirement. Insulin responsiveness, as estimated by the carbohydrate to insulin ratio (C/I) and insulin sensitivity factor (ISF), was found to decrease significantly with increasing Tanner stage (*P* for linear trend = 0.001 for C/I and ISF, resp., Table 3).

**Figure 2:**
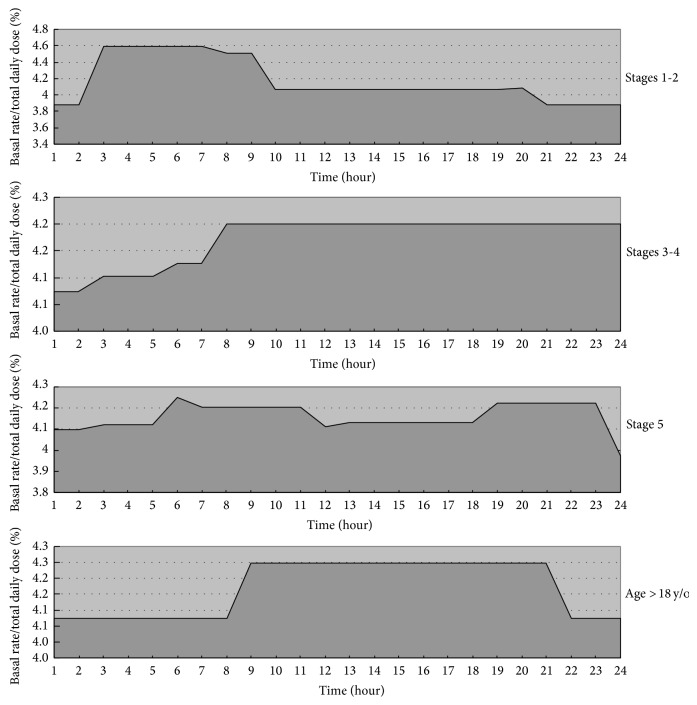
Circadian pattern of basal insulin use in different groups. The *y*-axis represents the percentage of basal insulin rate divided to total daily insulin dose (basal insulin rate/total daily dose ×100%).

As illustrated in Figure 2, the daily pattern of basal insulin use differed markedly between the five Tanner stages groups. Specifically, basal insulin requirement was highest between 3:00 and 7:00 h among patients in Tanner stages 1-2. By comparison, lower nocturnal basal insulin which then increased gradually until day time was noted among patients in stages 3-4. Adolescents (Tanner stage 5) displayed a high basal insulin peak between 6:00 and 11:00 h and a smaller peak between 19:00 and 23:00 h. In the young adult group (>18 years old), a lower basal insulin rate was present between 22:00 and 8:00 h.

## 4. Discussion

An insulin pump is an attractive option for intensive therapy at any age and has been found to improve patient quality of life [7–9]. The greater cost of the pump and associated supplies compared to that of syringes and needles used in MDI therapy has led to the conservative use of CSII [10]. Increasingly, CSII is being used as the first-line therapy at diabetes onset in preschool children; the use of insulin analogs has become standard in CSII [11]. However, there is lack of efficacy data on CSII in type 1 diabetic children in Taiwan. This is the first study in Taiwan to report the effectiveness of CSII in pediatric type 1 diabetes across different developmental stages.

The challenge of CSII involves the administration of the insulin dose and distribution of basal insulin. Depending on glycemic control, the total daily dose may be reduced by 10–20% or more in case of frequent hypoglycemia or high total insulin dose during MDI, then using 30–50% of total daily dose as daily basal rate [12, 13]. In this study, the basal insulin accounted for less than 40% of total daily insulin dose. A smaller proportion of basal insulin during CSII among Asian versus Western populations was also documented in our previous study of young adult patients with T1D [3]. Some other report had revealed the lower basal to total daily dose ratio in Japanese T1D whose food was similar to Taiwanese. Kuroda et al. demonstrated that the basal insulin requirement was about 30% of the total daily insulin dose in T1D patients who used the insulin pump [14]. But the ages of the participants were older (39.7 ± 10.9 years) than our current study. Therefore, the findings in the current study were still valuable in caring such a young-age population.

Although the ADA recommends blood glucose testing be conducted at least four times a day, more frequent monitoring may be required for CSII therapy in type 1 diabetic children [15]. In this study, the frequency of SMBG increased from four to six times per day after initiation of CSII. The educational effect and the behavior change brought about by CSII could lead to an improvement in diabetic control. In addition, since the weekly duration of exercise significantly increased following CSII, the CSII equipment is unlikely to prevent daily exercise. Instead, the reduced frequency of hypoglycemia in CSII and flexible basal insulin adjustment might encourage the patients to perform exercise more regularly [3].

Table 3 indicates that total insulin dosing was decreased by at least 10.8% across all Tanner stages following CSII. The adjusted proportion of basal to total daily demand was less than 40%. This is a relatively high bolus insulin proportion compared to the 50% reported in other studies [16–22]. Rice-based meal contents and low fatty food in the typical Taiwanese diet may be a possible explanation of this discrepancy [23]. Additionally, the decreased C/I ratio and ISF across development stages likely reflects the change in body composition of the insulin pump user. The weight gain of these patients could have some effect on the C/I and ISF. But the weight and development stage was highly associated as presented in Table 1. The meaningful correlation of development stage and insulin requirement could be the chief characteristic presentation in T1D patients. The difference in insulin requirement in prepubertal and pubertal children had also been proposed in previous studies [3, 18, 19, 21, 24, 25].

Age-dependent endocrine changes during childhood, particularly during puberty, affect the circadian pattern of insulin needs in CSII [26]. While the difference in circadian pattern of basal insulin in prepubertal and pubertal children had been proposed in previous studies [18, 21, 25], these study samples consisted mostly of Caucasians and were divided by age rather than Tanner stage. The varied approaches to age grouping lead to different patterns across studies [3, 18, 19, 21, 24, 25]. We demonstrated that the morning rise of basal dose of insulin, which reflects the emergence of the dawn phenomenon, can occur as early as at Tanner stages 1-2. The higher basal insulin during daytime at Tanner stages 3-4 represented the relative less dawn phenomenon in this development stage. By Tanner stage 5, two prominent insulin peaks, including one in the early morning and the other in the evening, that parallel the dawn-dusk pattern were identified. The relatively lower basal insulin in the daytime for these children might be partly explained by the highest children activity and vigorous exercises. After the age of 18 years, the basal rate pattern is close to the adult form with the lowest level during sleep. Through these analyses of different Tanner stages, we are better able to set and titrate the basal insulin dose as provided via CSII among type 1 diabetic children in Taiwan.

According to criteria of Tanner staging, Tanner stage 1 indicates prepuberty and Tanner stage 2 indicates early puberty. The pattern of insulin requirement was similar between the Tanner stages 1 and 2 in the current study. In addition, the number in Tanner stage 1 was only 3 with age between 9 and 12 years old, which was very close to stage 2 (age range 9–13 years old). For statistical reason, we pooled these stages together. Further large scale study may be needed to augment the results.

## 5. Conclusions

CSII therapy was effective for reducing A1c, increasing the frequency of SMBG and the duration of exercise in children and adolescents across different Tanner stages. The proportion of basal to total insulin use during CSII therapy was lower in this Taiwanese sample of children with type 1 diabetes by comparison with that reported for Western samples. Type 1 diabetic patients with higher pubertal stages were characterized by a decreased C/I ratio and ISF along with a different circadian pattern of insulin dosage.

## Figures and Tables

**Table 1:** Patient characteristics and daily insulin requirement by Tanner stage group before continuous subcutaneous insulin infusion (CSII) therapy.

	All stages	Stage 1 or 2	Stage 3 or 4	Stage 5	Young adult (>18 years old)	*P*
*n*	45	9	10	18	8	
Girl (%)	58	33	60	67	63	0.410
Height (cm)	155.7 ± 11.8	144.2 ± 8.3	149.4 ± 8.4	160.8 ± 8.4	165.4 ± 11.6	<0.001^*^
Height-SDS	0.24 ± 0.17	0.27 ± 1.02	0.36 ± 1.37	0.15 ± 0.97	0.25 ± 1.37	0.972
Weight (kg)	47.1 ± 10.9	37.4 ± 7.9	39.3 ± 5.9	50.5 ± 7.7	60.1 ± 7.7	<0.001^*^
Weight-SDS	−0.12 ± 0.68	−0.12 ± 0.99	−0.22 ± 0.85	−0.26 ± 0.33	0.29 ± 0.61	0.276
BMI (kg/m^2^)	19.2 ± 2.6	18.0 ± 3.1	17.5 ± 1.7	19.5 ± 1.9	22.0 ± 2.1	<0.001^*^
BMI-SDS	−0.24 ± 0.65	−0.24 ± 0.93	−0.43 ± 0.61	−0.34 ± 0.42	0.22 ± 0.70	0.156
T1D duration (years)	5.3 ± 4.1	3.7 ± 3.6	3.4 ± 3.0	5.9 ± 3.9	8.1 ± 4.9	0.050
HbA1c (mmol/mol)	67 ± 30	51 ± 8	79 ± 52	66 ± 26	73 ± 10	0.221
HbA1c (%)	8.3 ± 2.8	6.8 ± 0.8	9.3 ± 4.7	8.2 ± 2.4	8.9 ± 0.9	0.221

Data are mean ± standard deviation or percentage. ^*^Significant difference (*P* < 0.05) by ANOVA test.

**Table 2:** Clinical variables before and after continuous subcutaneous insulin infusion (CSII) therapy in all participants.

	3 months before	3 months after	1 year later	Last visit
HbA1c (mmol/mol)	67 ± 30	54 ± 17^*^	54 ± 17^*^	54 ± 11^*^
HbA1c (%)	8.3 ± 2.8	7.1 ± 1.5^*^	7.1 ± 1.0^*^	7.1 ± 1.0^*^
Fasting sugar (mmol/L)	9.8 ± 5.6	7.0 ± 2.9^*^	7.3 ± 3.1^*^	6.8 ± 2.5^*^
Triglyceride (mmol/L)	0.94 ± 1.65	0.62 ± 0.27	0.62 ± 0.28	0.55 ± 0.14
Triglyceride (mg/dL, log transformed)	1.79 ± 0.24	1.69 ± 0.13^*^	1.71 ± 0.16^*^	1.68 ± 0.11^*^
Cholesterol (mmol/L)	4.14 ± 0.75	4.06 ± 0.73	4.10 ± 0.71	4.18 ± 0.81
HDL-C (mmol/L)	1.64 ± 0.38	1.56 ± 0.34	1.55 ± 0.32	1.51 ± 0.31
LDL-C (mmol/L)	2.29 ± 0.78	2.33 ± 0.73	2.34 ± 0.72	2.42 ± 0.80
ALT (U/L)	12.1 ± 4.0	12.0 ± 4.1	11.6 ± 4.1	13.8 ± 8.8
Creatinine (*μ*mol/L)	44.0 ± 13.1	45.4 ± 10.7	45.1 ± 11.0	48.6 ± 9.8
SMBG (times/day)	3.8 ± 1.4	6.3 ± 1.6^*^	4.5 ± 1.4^*^	4.5 ± 1.4^*^
Exercise (minutes/week)	89.7 ± 35.6	130.6 ± 58.2^*^	164.0 ± 82.8^*^	164.0 ± 82.8^*^

Data are mean ± standard deviation or percentage. *P* for ANOVA test.

^*^Significant difference (*P* < 0.05) by paired *t*-test as compared to baseline data.

SMBG: self-monitoring of blood glucose.

**Table 3:** Insulin dose at continuous subcutaneous insulin infusion (CSII) therapy.

	All stages	Stage 1 or 2	Stage 3 or 4	Stage 5	Young adult (>18 years old)	*P*
Prepump TDD (U/kg)	1.13 ± 0.42	1.13 ± 0.37	1.00 ± 0.29	1.25 ± 0.53	1.01 ± 0.23	0.382
Postpump TDD (U/kg)	0.87 ± 0.22^*^	0.95 ± 0.23	0.85 ± 0.18	0.87 ± 0.25^*^	0.78 ± 0.14^*^	0.438
Chang of TDD (%)	20.8 ± 22.8	10.8 ± 22.5	21.1 ± 32.9	25.4 ± 19.4	21.6 ± 14.1	0.233
Basal insulin (U/kg/day)	0.31 ± 0.07	0.32 ± 0.08	0.34 ± 0.08	0.31 ± 0.07	0.27 ± 0.04	0.252
Basal insulin proportion (% of TDD)	36.5 ± 6.3	33.6 ± 4.7	39.6 ± 6.4	36.8 ± 6.3	35.5 ± 7.1	0.226
C/I	10.2 ± 4.2	12.9 ± 5.4	12.7 ± 4.8	9.0 ± 2.6	7.3 ± 1.5	0.001^**^
ISF	56.4 ± 13.6	66.7 ± 15.0	61.2 ± 13.5	52.5 ± 11.7	48.1 ± 8.0	0.001^**^

Data are mean ± standard deviation or percentage.

^*^Significant difference (*P* < 0.05) as compared with the prepump TDD by paired *t*-test (two-tailed).

^**^ *P* for linear trend <0.05 by ANOVA test.

TDD: total daily dose; C/I: carbohydrate to insulin ratio; ISF: insulin sensitivity factor.

## References

[1] Fisher L. K. (2006). The selection of children and adolescents for treatment with continuous subcutaneous insulin infusion (CSII). *Pediatric Diabetes*.

[2] Bachran R., Beyer P., Klinkert C., Heidtmann B., Rosenbauer J., Holl R. W. (2012). Basal rates and circadian profiles in continuous subcutaneous insulin infusion (CSII) differ for preschool children, prepubertal children, adolescents and young adults. *Pediatric Diabetes*.

[3] Lin C.-H., Huang C.-H., Tsai J.-S. (2011). Effects of a novel short-term continuous subcutaneous insulin infusion program evaluated by continuous glucose monitoring on young adult type 1 diabetic patients in Taiwan. *Endocrine Journal*.

[4] Marshall W. A., Tanner J. M. (1969). Variations in pattern of pubertal changes in girls. *Archives of Disease in Childhood*.

[5] Marshall W. A., Tanner J. M. (1970). Variations in the pattern of pubertal changes in boys. *Archives of Disease in Childhood*.

[6] Juang J.-H., Huang B.-Y., Huang H.-S., Lin J.-D., Huang M.-J. (1989). C-peptide response to glucagon in young diabetics. *Taiwan Yi Xue Hui Za Zhi*.

[7] Bergenstal R. M., Tamborlane W. V., Ahmann A. (2010). Effectiveness of sensor-augmented insulin-pump therapy in type 1 diabetes. *The New England Journal of Medicine*.

[8] Fox L. A., Buckloh L. M., Smith S. D., Wysocki T., Mauras N. (2005). A randomized controlled trial of insulin pump therapy in young children with type 1 diabetes. *Diabetes Care*.

[9] Weinzimer S. A., Swan K. L., Sikes K. A., Ahern J. H. (2006). Emerging evidence for the use of insulin pump therapy in infants, toddlers, and preschool-aged children with type 1 diabetes. *Pediatric Diabetes*.

[10] Plotnick L. P., Clark L. M., Brancati F. L., Erlinger T. (2003). Safety and effectiveness of insulin pump therapy in children and adolescents with type 1 diabetes. *Diabetes Care*.

[11] Berghaeuser M. A., Kapellen T., Heidtmann B., Haberland H., Klinkert C., Holl R. W. (2008). Continuous subcutaneous insulin infusion in toddlers starting at diagnosis of type 1 diabetes mellitus. A multicenter analysis of 104 patients from 63 centres in Germany and Austria. *Pediatric Diabetes*.

[12] Pańkowska E., Szypowska A., Lipka M. (2008). Basal insulin and total daily insulin dose in children with type 1 diabetes using insulin pumps. *Pediatric Diabetes*.

[13] Phillip M., Battelino T., Rodriguez H., Danne T., Kaufman F. (2007). Use of insulin pump therapy in the pediatric age-group: consensus statement from the European Society for Paediatric Endocrinology, the Lawson Wilkins Pediatric Endocrine Society, and the International Society for Pediatric and Adolescent Diabetes, endorsed by the American Diabetes Association and the European Association for the Study of Diabetes. *Diabetes Care*.

[14] Kuroda A., Kaneto H., Yasuda T. (2011). Basal insulin requirement is ∼30% of the total daily insulin dose in type 1 diabetic patients who use the insulin pump. *Diabetes Care*.

[15] Silverstein J., Klingensmith G., Copeland K. (2005). Care of children and adolescents with type 1 diabetes: a statement of the American Diabetes Association. *Diabetes Care*.

[16] Bode B., Weinstein R., Bell D. (2002). Comparison of insulin aspart with buffered regular insulin and insulin lispro in continuous subcutaneous insulin infusion: a randomized study in type 1 diabetes. *Diabetes Care*.

[17] Bolli G. B., Kerr D., Thomas R. (2009). Comparison of a multiple daily insulin injection regimen (basal once-daily glargine plus mealtime lispro) and continuous subcutaneous insulin infusion (lispro) in type 1 diabetes: a randomized open parallel multicenter study. *Diabetes Care*.

[18] Conrad S. C., McGrath M. T., Gitelman S. E. (2002). Transition from multiple daily injections to continuous subcutaneous insulin infusion in type 1 diabetes mellitus. *The Journal of Pediatrics*.

[19] Danne T., Battelino T., Kordonouri O. (2005). A cross-sectional international survey of continuous subcutaneous insulin infusion in 377 children and adolescents with type 1 diabetes mellitus from 10 countries. *Pediatric Diabetes*.

[20] Deiss D., Hartmann R., Hoeffe J., Kordonouri O. (2004). Assessment of glycemic control by continuous glucose monitoring system in 50 children with type 1 diabetes starting on insulin pump therapy. *Pediatric Diabetes*.

[21] Klinkert C., Bachran R., Heidtmann B., Grabert M., Holl R. W. (2008). Age-specific characteristics of the basal insulin-rate for pediatric patients on CSII. *Experimental and Clinical Endocrinology and Diabetes*.

[22] Shashaj B., Sulli N. (2009). Difference in insulin usage patterns with pubertal development in children with type 1 diabetes during transition from multiple daily injections to continuous subcutaneous insulin infusion (CSII) and through the CSII treatment. *Diabetes Technology & Therapeutics*.

[23] Taiwan Ministry of Health and Welfare (2013). *The Database of Food and Nutrition in Taiwan*.

[24] Scheiner G., Boyer B. A. (2005). Characteristics of basal insulin requirements by age and gender in Type-1 diabetes patients using insulin pump therapy. *Diabetes Research and Clinical Practice*.

[25] Szypowska A., Lipka M., Błazik M., Golicka D., Groele L., Pańkowska E. (2009). Age-dependent basal insulin patterns in children with type 1 diabetes treated with continuous subcutaneous insulin infusion. *Acta Paediatrica*.

[26] Holterhus P.-M., Odendahl R., Oesingmann S. (2007). Classification of distinct baseline insulin infusion patterns in children and adolescents with type 1 diabetes on continuous subcutaneous insulin infusion therapy. *Diabetes Care*.

